# Transurethral en bloc resection of a bladder perivascular epithelioid cell tumor (PEComa): a case report

**DOI:** 10.1186/s12894-023-01198-6

**Published:** 2023-03-02

**Authors:** Shi-Ping Zeng, Yi-Fei Sun, Jun-Bing Ye, Ke Zeng, Xiao-Bin Li

**Affiliations:** 1 Department of Urology, Zigong First People’s Hospital, Zigong, China; 2 Neurological Disease Laboratory, West China Hospital of Sichuan University, Chengdu, China

**Keywords:** Perivascular epithelioid cell tumor, PEComa, Bladder, Transurethral en bloc resection, ERBT

## Abstract

**Background:**

Perivascular epithelioid cell tumor (PEComa) is a mesenchymal tumor with distinct histologic and immunologic features. PEComas that originate in the bladder are extremely rare clinically, with only 35 cases reported in the English literature thus far. Here, we report a case of bladder PEComa resection by transurethral en bloc resection of bladder tumor (ERBT).

**Case presentation:**

A 66-year-old female with a history of poorly controlled type 2 diabetes with associated complications of frequent urinary tract infections presented to our hospital for a routine physical examination. Outpatient ultrasound examination revealed a strong echogenic mass of approximately 1.5 × 1.3 × 1.3 cm in size on the posterior wall of the bladder. The enhanced computed tomography and enhanced magnetic resonance imaging after admission both suggested a well-defined isolated nodular mass on the posterior wall of the bladder with significant enhancement on the enhanced scan. The tumor was successfully and completely resected by ERBT. Postoperative pathological examination and immunohistochemical results confirmed the mass was a bladder PEComa. No tumor recurrence was observed in the six-month postoperative follow-up.

**Conclusion:**

Bladder PEComa is an extremely rare mesenchymal tumor of the urinary system. When imaging and cystoscopy reveal a nodular mass with an abundant blood supply in the bladder, PEComa should be included in the differential diagnosis of bladder tumors. Surgical resection is currently the primary option for the treatment of bladder PEComa. For a solitary, pedunculated, narrow-based, small-sized bladder PEComa, resection of the tumor by ERBT was a safe and feasible approach in our patient and may be considered for similar cases in the future.

## Background

Perivascular epithelioid cell tumor (PEComa) is a mesenchymal tumor with distinct histologic and immunologic features. The tumor is composed of a mixture of epithelioid and spindle cells and is usually immunologically expressed with both melanocytic and myoid markers [1]. Some PEComas also have TFE3 gene rearrangements [2]. The PEComa family includes angiomyolipomas, lymphangioleiomyosarcomas, clear-cell sugar tumors, and other perivascular epithelioid cell-derived tumors [3, 4]. In the urinary system, PEComa is most commonly found in the kidney but is extremely rare in the bladder, prostate, testis, and urethra [5]. Only 35 cases of bladder PEComa have been reported in the English literature thus far. In this paper, we report a case of bladder PEComa resected by transurethral en bloc resection of bladder tumor (ERBT).

## Case presentation

**Fig. 1:**
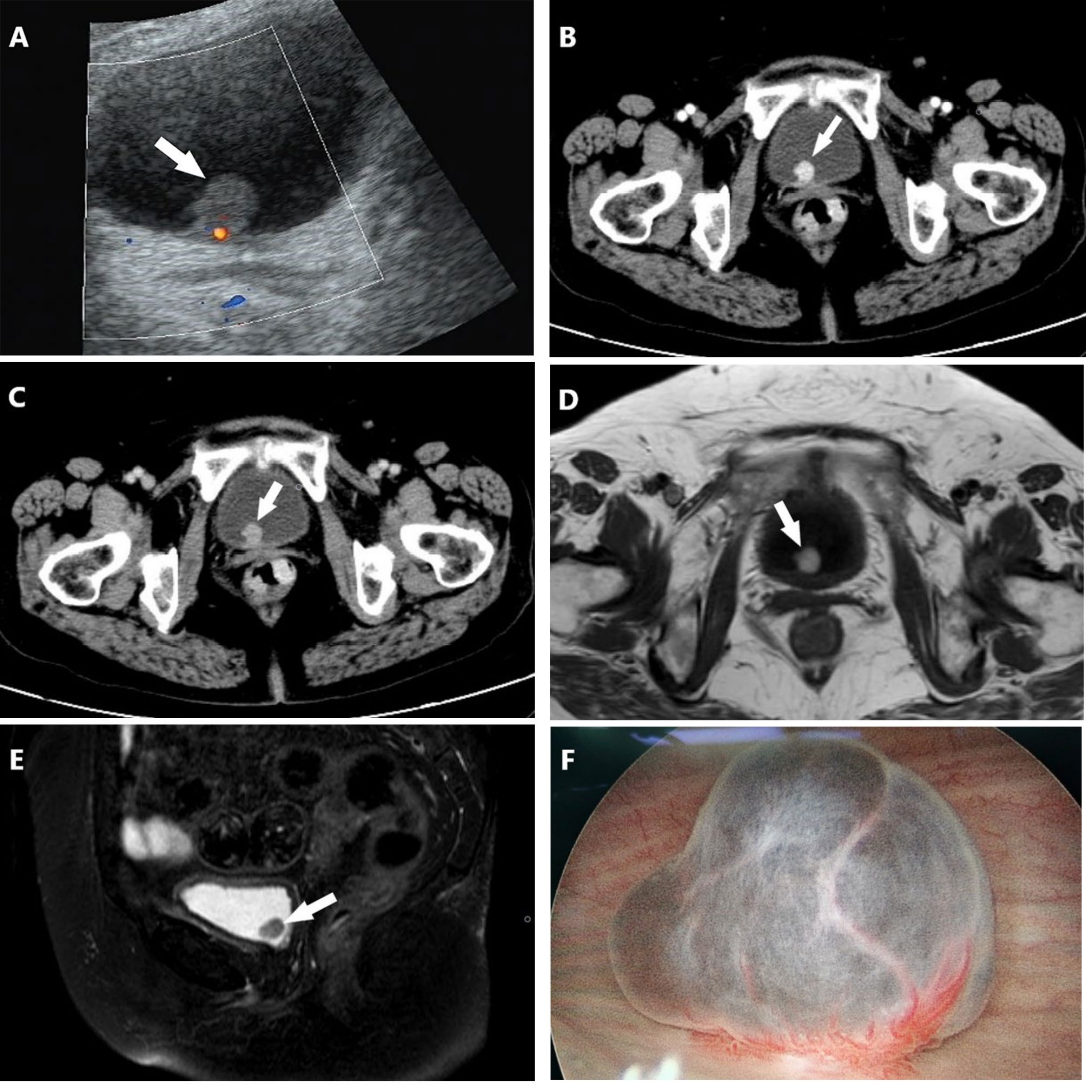
(A) Ultrasound suggested a neoplastic lesion (white arrow) approximately 1.5 × 1.3 cm in size with a regular morphology and clear borders on the posterior wall of the bladder, in which blood flow signals were visible. (B, C) Contrast-enhanced CT scan axial images suggested a round mass (white arrow) with a maximum diameter of approximately 1.4 cm on the posterior wall of the bladder, with clear borders and obvious enhancement. (D, E) MRI axial T1WI and sagittal T2WI suggested a nodular mass (white arrow) with a maximum diameter of approximately 1.3 cm on the posterior wall of the bladder with clear borders and a high signal on T1WI and T2WI-SPAIR. (F) Cystoscopically, the tumor was observed to be round and reddish-brown, with a pedicle and a large number of tortuous vessels at the base

**Fig. 2:**
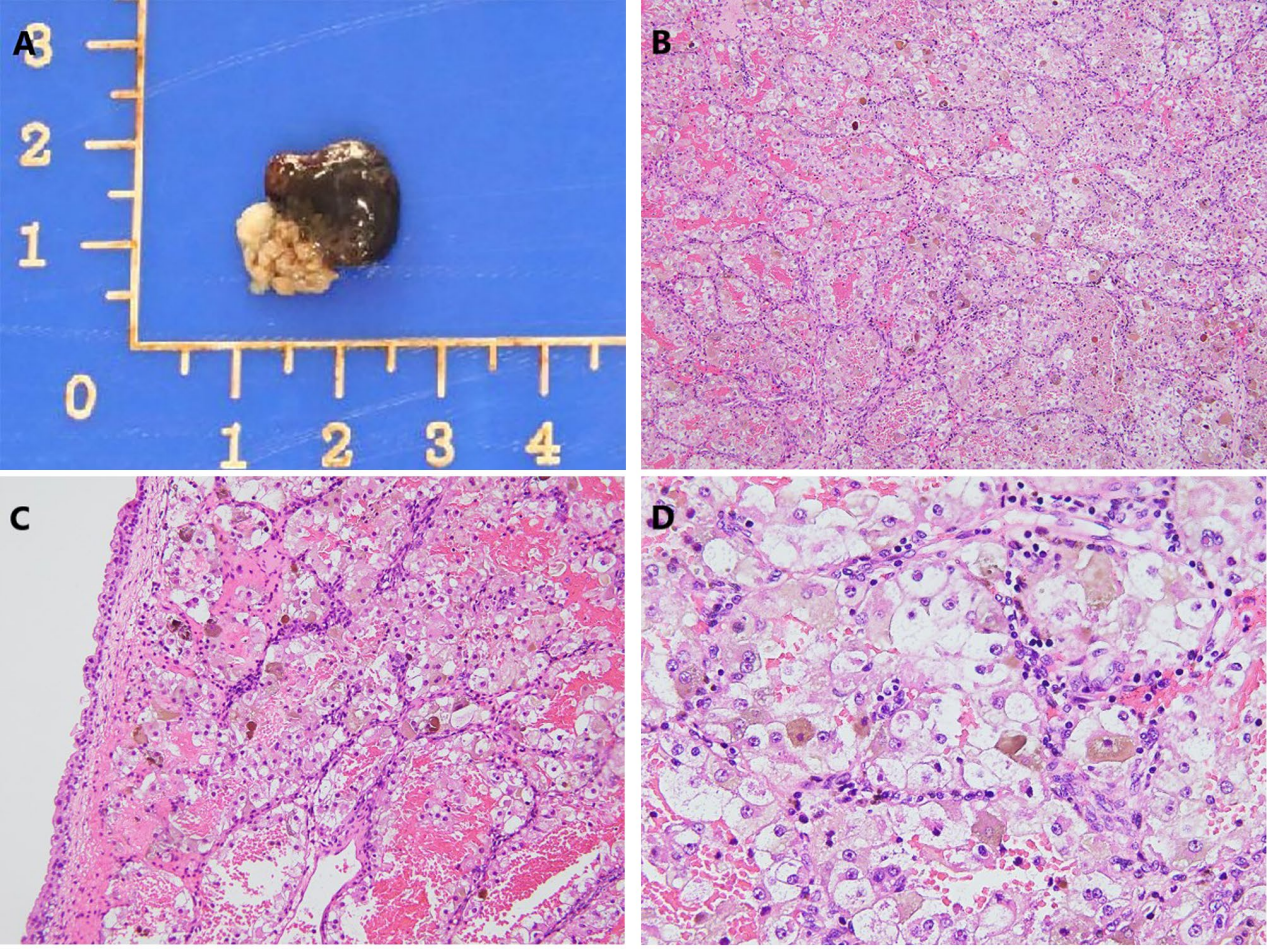
(A) The tumor specimen was round, approximately 1.3 cm in size, reddish-brown, solid, with a smooth surface, and a tumor base that was preserved intact. (B, C) The tumor consisted of epithelioid cells arranged in a nested or glandular pattern with abundant interstitial vascularity (H&E × 100, H&E × 200). (D) The nuclei of the tumor cells had mild heterogeneity with visible nucleoli and clear or eosinophilic cytoplasm (H&E × 400)

A 66-year-old female with a history of poorly controlled type 2 diabetes with associated complications of frequent urinary tract infections presented to our hospital for a routine physical examination. On examination, she complained of repeated urinary tract infections for the past 3 years and asked for a review of the urinary system for abnormalities, prompting an ultrasound to be completed. Ultrasound showed a strong echogenic mass of approximately 1.5 × 1.3 × 1.3 cm in size on the posterior wall of the bladder, with regular morphology and clear borders, and color Doppler flow imaging (CDFI) showed the presence of a blood flow signal in the lesion (Fig. 1A). The patient was admitted to the urology department. Urinalysis after admission revealed white blood cells: 17.48/hp, bacterial count: 39,260/µl, urine occult blood: 2+, and glucose: 2+. The rest of the laboratory tests showed no abnormalities. A contrast-enhanced CT scan showed a round nodular mass on the posterior wall of the bladder with clear borders and a maximum diameter of approximately 1.4 cm (Fig. 1B, C). MRI suggested a nodular mass with a maximum diameter of approximately 1.3 cm on the posterior wall of the bladder, with clear borders, high signal on T1WI, T2WI-SPAIR and DWI, and a low signal on ADC. The mass was significantly enhanced on MRI-enhanced scans, the inner layer of the bladder wall adjacent to the mass was also enhanced, and the muscular layer of the bladder wall was not enhanced (Fig. 1D, E). The patient then underwent cystoscopy, which showed a round mass of approximately 1.3 × 1.2 cm in size, reddish-brown in color, with a pedicle and a copious basal blood supply, on the right side of the posterior bladder wall (Fig. 1F). Due to concerns about post-biopsy mass bleeding, mass biopsy was not performed. The patient eventually underwent diagnostic ERBT. A SIMAI bipolar plasma system and bipolar loop electrode (SIMAI: SM-8100) were used in the surgery. The cutting and coagulation power were adjusted to 60 and 80 W, respectively. First, we marked and blocked the blood supply to the tumor area by electrocoagulation with an electrode at a distance of 5 mm from the ring of the tumor edge and then incised, inserted, and provoked the mucosal layer and submucosal layer tissues of the bladder wall with the tip of the ring electrode. Then, we incised the muscle layer tissues to the deep muscle layer, peeled the tumor along the plane of the deep muscle layer by pushing flatly, separated the interlaced muscle fiber bundles in this plane, and carefully stopped the bleeding point. The tumor was successfully removed during surgery, and its integrity was preserved. The tumor specimen was round, reddish-brown, solid, with a smooth surface (Fig. 2A). Postoperative pathological examination suggested that the tumor consisted of epithelioid cells arranged in a nested or glandular pattern, with mild heterogeneity of the nucleus, visible nucleoli, clear or eosinophilic cytoplasm, abundant interstitial vessels, clear tumor boundaries, and tumor infiltration of the lamina propria of the bladder wall (Fig. 2B-D). Immunohistochemical staining results were as follows: CK (-), Vimentin (-), GATA3 (-), S100 (-), HMB45 (partial +), Melan-A (partial +), CD31 (vascular +), CD34 (vascular +), PAX8 (-), Ki-67 (5% +). The pathological examination and immunohistochemical results indicated a final diagnosis of the mass as bladder PEComa. Since the tumor did not infiltrate the muscle layer of the bladder wall, the patient did not undergo further surgery and did not receive intravesical chemotherapy. The patient had clear postoperative indwelling catheter drainage without bladder irrigation, and the catheter was successfully removed 5 days after surgery. She underwent cystoscopy 3 months after surgery and CT examination 6 months after surgery, and no tumor recurrence was found.

## Discussion and conclusions

PEComa is a mesenchymal tumor composed of histologically and immunologically distinctive perivascular epithelioid cells [4]. The PEComa family includes angiomyolipomas, lymphangioleiomyosarcomas, clear-cell sugar tumors, and other perivascular epithelioid cell-derived tumors [3, 4]. PEComa has been reported in many organs and tissues of the body, such as the lung, liver, pancreas, breast, uterus, vulva, ovary, broad ligament, heart, skull base, and soft tissues [6, 7]. In the urinary system, PEComa is most commonly found in the kidney, while PEComa originating in the bladder is extremely rare.

Patients with bladder PEComa tend to be young to middle-aged and more often women than men [1]. The majority of patients present with hematuria, while some may also exhibit symptoms of abdominal discomfort, abdominal pain, abdominal distension, difficulty in urination, urinary frequency, urinary urgency, etc. There are also a few patients who do not have any clinical symptoms and are found incidentally by examination [1, 8]. Previous reports have suggested an association between PEComa and tuberous sclerosis (TSC). However, only two cases of bladder PEComa have thus far presented typical clinical features of TSC, such as shagreen patches and cutaneous angiofibromas [8–10]. The imaging features of bladder PEComa are not specific. Ultrasonography often shows a well-defined ovoid, nodular, hypoechoic, or isoechoic solid mass; CDFI shows abundant blood flow signals within and around the lesion [1]. Unenhanced CT scanning shows a nodular, irregular, well-defined solid or cystic mass with homogeneous or heterogeneous density and necrosis within the mass [1, 11]. On contrast-enhanced CT scans, the mass shows obvious uneven enhancement in the arterial phase and rapid washout of the contrast in the venous phase. The solid part of the tumor is weakly enhanced in the delayed phase, the liquid part is not enhanced, and the marginal part is circumferentially enhanced [4, 11]. On MRI, the mass shows an isosignal on T1WI and a heterogeneous high signal on T2WI [4, 11].

On cystoscopy, bladder PEComa presents as a nodular or polypoid mass with or without a pedicle. The tumors are mostly located on the right and left lateral walls of the bladder and are often solitary lesions [1, 5]. Grossly, the tumor is often spherical or nodular, with clear borders and a firm appearance. The cut surface of the tumor is grayish-white or grayish-yellow with focal cystic regions, occasional hemorrhage, and necrosis [1, 8]. The tumor may invade the inner layer and muscular propria of the bladder wall and even the extravesical tissues [1]. Pathologically, most bladder PEComas consist of a mixture of epithelioid cells and spindle cells arranged in bundles, trabeculae, sheets, or nests [1, 8]. Epithelioid cells often have round-to-ovoid nuclei with small nucleoli and occasional nuclear inclusions; the cytoplasm is abundant, hyaline to granular, and eosinophilic [6, 8]. Spindle cells are spindle-shaped with elongated nuclei, similar to the characteristics of muscle cells [6]. The mitotic activity of the tumor cells was low, with 1–3 mitoses/HPF [5]. Histological variants of these cells include stromal hyalinization, myxoid changes, stromal microcysts, and multinucleated cells. Thin-walled and/or glassy vessels and occasionally larger vessels can be seen between the nests of tumor cells [8]. Folpe et al. classified PEComas into three categories based on their histologic features: benign, uncertain malignant potential, and malignant. They proposed criteria associated with the malignant behavior of PEComas, including tumor size > 5 cm, infiltration, high nuclear grade and cellularity, mitotic rate ≥ 1/50 HPF, necrosis, and vascular invasion. Tumors with two or more of these characteristics are considered to be predisposed to malignancy [12].

The literature data show that bladder PEComa has a relatively consistent immunophenotype, with tumor cells often coexpressing melanocytic and myoid markers. All tumor cells express the melanocytic marker HMB45, but the expression rate of Melan-A is only 31.8%, and that of SMA is 78.6%. Some tumor cells express Desmin, Calponin HHF35, CD34, S100, CD117, or Smooth muscle myosin heavy chain, but CK, Vimentin, Muscle-specific actin, Myoglobin, PAX8, CD31, WT-1, and EMA are often negatively expressed [1, 6]. Based on immunohistochemical results, bladder PEComa needs to be differentiated from other bladder malignancies (including epithelioid sarcoma, paraganglioma, smooth muscle sarcoma, inflammatory myofibroblastoma, extragastric mesenchymal tumor, melanoma, clear cell sarcoma of soft parts and metastatic tumors) [6, 8].

Molecularly, the transcription factor binding to IGHM enhancer 3 (TFE3) and its protein, located on the short arm of chromosome Xp11.23, belong to the microphthalmia-associated transcription (MIT) family [13]. A recent study found that 23% of PEComas have TFE3 gene rearrangements [2]. The first case of bladder PEComa with a TFE3 gene rearrangement was reported by Folpe et al. in 2005 [14]. The characteristics of bladder PEComa with TFE3 translocation reported by Russell and Williamson et al. were tumor size > 4 cm, moderate-to-high heterogeneity, necrosis, mitosis > 1/50 HPF, presence of vascular invasion, late stage of disease, and poor prognosis [15, 16]. However, Vannucchi and Chen et al. reported that the characteristics of bladder PEComa with TFE3 translocation were tumor size < 4 cm, mild heterogeneity, absence of necrosis and vascular invasion, mitosis ≤ 1/50 HPF, and good prognosis [13, 17]. Therefore, the relationship between TFE3 rearrangement and the clinicopathological characteristics and prognosis of bladder PEComa needs to be further investigated.

There is no definitive conclusion about the best treatment options for bladder PEComa. Surgical resection is still the primary treatment modality, which includes transurethral resection of bladder tumor (TURBT), partial cystectomy, and radical cystectomy. TURBT is mostly used for patients whose tumors have invaded the submucosal and lamina propria layers of the bladder wall. Partial cystectomy and radical cystectomy are suitable for patients whose tumors have invaded the muscular layer of the bladder wall or even the whole bladder wall or as a complementary treatment when TURBT cannot completely remove the tumor [1, 16, 18, 19].

TURBT should be carefully chosen to remove tumors with a rich blood supply or large size. Russell et al. reported a patient with bladder PEComa who underwent TURBT. During the operation, it was found that the tumor was rich in blood supply, there was heavy bleeding during tumor resection, and it was difficult to stop the bleeding. Therefore, the patient eventually underwent a partial cystectomy [15]. You-li et al. reported two patients presenting bladder PEComa with tumor diameters of 5.5 and 6 cm, respectively, who underwent TURBT; despite complete surgical resection, tumor recurrence occurred 10 and 13 months after surgery, respectively [1]. In the present case report, we performed ERBT to remove the tumor, as it was a small and single mass. The surgery achieved complete tumor resection and obtained a complete tumor specimen through the urethra with little intraoperative bleeding and no surgery-related complications. Previous literature has reported that ERBT has the advantages of a lower surgical complication rate and higher quality of tumor specimens compared to conventional TURBT in bladder urothelial tumor surgery [20, 21]. We believe that for nonmuscle invasive bladder PEComas, especially solitary, pedunculated, narrow-based, small-sized tumors, ERBT is a good surgical approach to resect the mass to maintain tumor integrity, reduce intraoperative bleeding, and minimize tissue remnants. However, more studies are still needed to demonstrate the applicability of ERBT to bladder PEComa. For large bladder PEComas, partial cystectomy is perhaps more appropriate. In previous reports, partial cystectomy has also shown great advantages in controlling intraoperative bleeding and preserving tumor integrity. Wu et al. even suggested partial cystectomy as the preferred treatment for bladder PEComa, except for small polypoid masses with a pedicle [1].

Although most currently reported cases of bladder PEComa exhibit benign biological behavior, there have been a few reports of recurrence, metastasis, and even death from such tumors. Palleschi et al. reported a case of a patient with bladder PEComa in which the tumor had metastasized to the L1 and L5 vertebrae and the left iliac wing by the time of initial diagnosis [22]. Williamson et al. reported a patient with bladder PEComa who developed multiple abdominal metastases 10 months after radical cystectomy and eventually died 12 months after surgery [16]. Clinical scholars have made various attempts to treat advanced bladder PEComa. Palleschi et al. reported a patient with bladder PEComa with bone metastases who underwent endoscopic surgical resection of the tumor combined with gemcitabine-based systemic chemotherapy, and this patient achieved a good prognosis [22]. Parfitt et al. reported a patient with bladder PEComa with small bowel metastases and an entero-vesical fistula who underwent partial cystectomy and partial small bowel resection followed by interferon-alpha immunotherapy; unfortunately, no follow-up was performed [23].

Overall, bladder PEComa is an extremely rare mesenchymal tumor of the urinary system with distinct histologic and immunologic features. When imaging and cystoscopy reveal a nodular mass with an abundant blood supply in the bladder, PEComa should be included in the differential diagnosis of bladder tumors. Surgical resection is currently the primary option for the treatment of bladder PEComa. For a solitary, pedunculated, narrow-based, small-sized bladder PEComa, resection of the tumor by ERBT was a safe and feasible approach in our patient and may be considered for similar cases in the future.

## Data Availability

All relevant data and materials are included in this article.

## Abbreviations

PEComa: Perivascular epithelioid cell tumor
ERBT: Transurethral en bloc resection of bladder tumor
CDFI: Color Doppler flow imaging
CT: Computed tomography
MRI: Magnetic resonance imaging
T1WI: T1-weighted image
T2WI: T2-weighted image
DWI: Diffusion-weighted imaging
ADC: Apparent diffusion coefficient
DCEI: Dynamic enhanced imaging
TSC: Tuberous sclerosis
HPF: High power field
TFE3: Transcription factor binding to IGHM enhancer 3
MIT: Microphthalmia-associated transcription
TURBT: Transurethral resection of bladder tumors.
